# Long-term mental health change patterns in ICU survivors: a four-year comparative follow-up from the SMAP–HoPe study

**DOI:** 10.1186/s40560-025-00812-z

**Published:** 2025-07-28

**Authors:** Takeshi Unoki, Tomoki Kuribara, Sakura Uemura, Mayumi Hino, Masako Shirasaka, Yuko Misu, Takumi Nagao, Mio Kitayama, Junpei Haruna, Masahiro Yamane, Keiko Atsumi, Miyuki Sagawa, Yumi Kajiyama, Kazuyuki Okada, Tomomi Nishide, Emiko Higuchi, Hideaki Sakuramoto

**Affiliations:** 1 Department of Acute and Critical Care Nursing, School of Nursing, Sapporo City University, Kita 11 Nishi 13, Chuo-Ku, Sapporo, 060-0011 Japan; 2 Emergency and Critical Care Medical Center, Osaka City General Hospital, 2-13-22 Miyakojima-Hondori, Miyakojima-Ku, Osaka, 534-0021 Japan; 3 Intensive Care Unit, Tohoku Medical and Pharmaceutical University Hospital, 1-12-1 Fukumuro, Miyagino-Ku, Sendai, 983-8512 Japan; 4 Intensive Care Unit & Cardiac Care Unit, Japanese Red Cross Fukuoka Hospital, 3-1-1 Ogusu, Minami-Ku, Fukuoka, 815-8555 Japan; 5 High Care Unit, Jichi Medical University Hospital, 3311-1, Yakushiji, Shimotsuke, 329-0431 Japan; 6 Intensive Care Unit, Sakakibara Heart Institute, Asahi-Cho, Fuchu, Tokyo 183-0003 Japan; 7 Department Heart Center, Kanazawa Medical University Hospital, 1-1, Uchinada, Kahoku, 920-0293 Japan; 8 Intensive Care Unit, Sapporo Medical University Hospital, Minami-1, Nishi-16, Chuo-Ku, Sapporo, 060-8543 Japan; 9 Intensive Care Unit, Jichi Medical University Hospital, 3311-1, Yakushiji, Shimotsuke, 329-0431 Japan; 10 Department of Adult Health Nursing, College of Nursing, Ibaraki Christian University, Omika 6-11, Hitachi, 319-1295 Japan

**Keywords:** Intensive care unit, Post-intensive care syndrome, Mental health disorder, Follow-up study

## Abstract

**Background:**

Post-intensive care syndrome (PICS) encompasses persistent physical, cognitive, and psychological impairments in individuals following intensive care unit (ICU) discharge. The short-term mental health impacts of PICS have been previously examined; however, long-term change pattern remain inadequately understood. In this study, we aimed to determine the prevalence of mental health disorders in individuals at 4 years post-ICU discharge, compare prevalence rates between 1 and 4 years, and identify change patterns and associated factors.

**Methods:**

In this 4-year follow-up study of the SMAP–HoPe study (754 ICU survivors from 12 Japanese ICUs were originally examined in the SMAP–HoPe study), we included participants from seven ICUs who completed mental health assessments using the Hospital Anxiety and Depression Scale and Impact of Event Scale-Revised at both 1- and 4-years post-ICU discharge. Growth mixture modeling was employed to identify distinct change patterns for anxiety, depression, and post-traumatic stress disorder (PTSD).

**Results:**

Among the 319 eligible participants, 223 (70.0%) provided responses. The prevalence of depression significantly increased from 24.7% at 1 year to 32.7% at 4 years (*p* = 0.021), whereas that of anxiety increased from 15.3% to 21.6% (*p* = 0.049). PTSD prevalence decreased from 5.1% to 2.7% (*p* = 0.549). Distinct change patterns were observed for anxiety—minimal (scores < 4) and decreasing, mild (scores ≥ 4) and increasing, and moderate (scores ≥ 8) and stable; for depression—minimal (scores < 4) and stable, mild (scores ≥ 4) and increasing, and moderate (scores ≥ 8) and stable; and for PTSD—minimal (scores < 4), mild (scores ≥ 4), and moderate (scores ≥ 10) symptoms that remained stable. Participants with higher education had a lower risk of exhibiting the moderate-stable depression change patterns (adjusted odds ratio: 0.25, 95% confidence interval: 0.09–0.68, *p* = 0.006).

**Conclusions:**

Mental health disorders in ICU survivors persist for a long term, with the prevalence of depression increasing over 4 years. Multiple change patterns were observed for each disorder, suggesting various progression courses. Participants with high education were protected from severe depression and its change patterns. These findings highlighted the importance of extended follow-up care and individualized interventions based on the change patterns and associated predictors.

**Supplementary Information:**

The online version contains supplementary material available at 10.1186/s40560-025-00812-z.

## Background

Post-intensive care syndrome (PICS) is a spectrum of physical, cognitive, and psychological impairments that persist following a patient’s stay in the intensive care unit (ICU), which significantly impact their quality of life (QOL) and social functioning after discharge [1]. Psychological disorders such as depression, anxiety, and post-traumatic stress disorder (PTSD) are particularly prevalent, posing a long-term burden on patients [2]. Advances in ICU care have contributed to improved survival rates among critically ill patients; however, this progress has also increased the prevalence of PICS. Thus, post-ICU mental health care should be prioritized in healthcare systems.

The long-term mental health impact of PICS has typically been examined in short-term studies, where patients were followed up for 0.5–2 years post-ICU [3–6]. However, only a few studies have reported the need for extended follow-up. Bienvenu et al. [7], in their 5-year longitudinal study involving patients recovering from acute respiratory distress syndrome (ARDS), found that psychiatric symptoms persisted long after ICU discharge, emphasizing the importance of understanding PICS-related mental health outcomes over longer periods. These findings suggest that longer follow-up periods are required to completely understand PICS. However, studies in which mental health was investigated over extended periods remain exceedingly limited. Therefore, tracking the long-term mental health of patients after ICU discharge is essential.

In previous studies, mental health severity was reported to follow distinct trajectories over time [8, 9]. For instance, among patients with the same mental health condition, some may show improvement, others may experience deterioration, and some maintain stable symptoms. Healthcare providers can develop more individualized follow-up approaches when these trajectories and their underlying factors are identified. However, studies where patients with mental health disorders were followed up over a long period are rare. Therefore, it is important to clarify these issues.

Hence, this study had three objectives—to determine the prevalence of mental health disorders (depression, anxiety, and PTSD) in patients at 4 years post-ICU discharge, to evaluate the long-term impact of PICS on patients’ mental health by comparing the prevalence rates between 1- and 4-years post-ICU discharge, and to identify change patterns in mental health severity between these time points and examine factors associated with these trajectories. Japan has one of the highest populations of older adults in the world. In the future, when other countries face aging societies similar to Japan's situation, findings from this research are expected to offer insight into what lies ahead for countries worldwide.

## Methods

### Study design

This study is a follow-up of the bidirectional cohort multicenter study, known as the SMAP–HoPe study [6]. In the SMAP–HoPe study, 754 survivors who had stayed in 12 ICUs for over three nights and were living at home 1 year after ICU discharge were examined. In that study, mental health outcomes, including depression symptoms, anxiety, and PTSD, were assessed 1 year after ICU discharge. For the present follow-up study, 7 of the 12 ICUs that participated in the follow-up phase of the SMAP–HoPe study were included. From these seven facilities, we focused on patients from the original study and reassessed their mental health, including symptoms of depression, anxiety, and PTSD, 4 years after ICU discharge, using the same evaluation methods that were employed in the SMAP–HoPe study. We used data collected 4 years after ICU discharge and compared it with the 1-year data from the SMAP–HoPe study. The original SMAP–HoPe study was conducted between October 2019 and July 2020. Accordingly, this follow-up study was conducted between October 2022 and July 2023.

The present study was reported in accordance with the Strengthening the Reporting of Observational Studies in Epidemiology Statement [10].

### Previous study (SMAP–HoPe study)

In the previous study, patients who had been discharged from the ICU 12 months earlier were retrospectively identified [6]. Furthermore, their health was prospectively evaluated through a mail survey in 12 Japanese ICUs. Participants were eligible if they had stayed in the ICU for at least three nights and had been living at home for 1 year post-discharge. Patients were excluded if they had central nervous system disorders such as stroke, traumatic brain injury, cerebral tumors, severe cognitive impairment, ICU readmission within 12 months, or if they had been transferred directly to another hospital. Additionally, those who were unable to complete the questionnaire, could not be reached by phone, or declined participation were not included. The patients were screened monthly using medical records. A recruitment letter was sent to inform them that a research nurse would contact them within a few days. The nurse called to verify eligibility and provided a brief explanation of the study, although formal consent was not obtained at this stage. We excluded those who refused or could not be reached after three attempts. Those who met the inclusion criteria received a survey package containing an explanatory leaflet and study questionnaires. The survey included the Impact of Event Scale-Revised (IES-R) [11], the Hospital Anxiety and Depression Scale (HADS) [12], and the EuroQOL-5 Dimension (EQ-5D-5L) [13]. Additionally, patient characteristics, delirium episodes during ICU admission, and hospital outcomes were retrospectively gathered from medical records. The study’s response rate was 91.1%.

### Recruitment process of the present study

Of the 12 ICUs that participated in the SMAP–HoPe study, seven facilities were included in this study. Originally, the research was to be implemented at 12 institutions; however, owing to the reassignment of principal investigators from the ICU to other departments at five sites, the study was eventually conducted across seven facilities.

We included participants from the SMAP–HoPe study who were treated at one of these seven facilities. Participants who died, were not living at home, were unable to complete a self-administered questionnaire, or had an unknown mailing address were excluded. Before sending out the survey questionnaires, we mailed postcards to confirm whether the participants met the exclusion criteria. Additionally, researchers reviewed the medical records of the participants from the SMAP–HoPe study to identify patients who had died or were hospitalized at the time of recruitment for this follow-up study. These patients were classified as meeting the exclusion criteria. All other eligible patients received a mail of the IES-R and HADS surveys. This process was repeated monthly to ensure that patients received the questionnaire in the fourth year after ICU discharge. If no response was received within 2 weeks, a single reminder was sent by post. If there was still no response after an additional 2 weeks, the case was classified as a nonresponder. This study was approved by the Research Ethics Committee of Sapporo City University (approval number: 2212-1). We provided explanatory documents and consent forms along with each survey set to the enrolled patients. Participants were required to mark a checkbox on the consent form to confirm their understanding of the study details and their agreement to participate.

### Variables and instruments

We used patient characteristics, treatment, and outcomes, such as ICU length of stay, which were obtained from the SMAP–HoPe study. The Acute Physiology and Chronic Health Evaluation II (APACHE II) and Sequential Organ Failure Assessment (SOFA) scores were calculated based on data collected from patients within 24 h of ICU admission. Sepsis was classified following the Sepsis-3 criteria [14]. Benzodiazepine exposure was defined as a continuous intravenous infusion in the ICU lasting for a minimal of 24 h. Malignancy was defined as the presence of active cancer during the ICU stay, based on diagnoses documented in the medical record.

Delirium assessments were routinely performed by nurses in all participating ICUs at least three times a day, using the Confusion Assessment Method for the ICU [15], the Intensive Care Delirium Screening Checklist [16], or both. Similarly, the data from the Richmond Agitation-Sedation Scale (RASS) [17] were systematically evaluated across all ICUs. A calendar day was considered “delirium present” if any assessment on that day were positive.

Coma was defined according to the RASS. A day was classified as “coma present” if all RASS assessments on that calendar day were consistently below –3. Although the exact frequency of RASS evaluations was unavailable due to the retrospective nature of the data, RASS scores were routinely assessed in daily ICU practice and documented in the medical records. The cumulative number of days with delirium and coma during the ICU stay was recorded for each patient.

All these data were meticulously extracted from medical records and were collected as part of the SMAP–HoPe study. Higher education was defined as having completed a university degree or higher.

Mental status was assessed using the unmodified Japanese versions of the IES-R [18] in the SMAP–HoPe study for PTSD screening and the HADS [19] for screening depression and anxiety in the present study. Detailed descriptions of these scales, including scoring methods, subscales, and psychometric properties, are provided in Additional file 1.

### Sample size calculation

This study is a follow-up study to our previous study [6], with a sample size dependent on both our previous study's sample size and response rate. Therefore, we conducted a post-hoc power analysis for 215 participants who completed the IES-R measurement, representing the smallest dataset obtained. Using a power of 80% (power = 0.8) and a significance level of 5% (*p* = 0.05), we calculated an effect size of *d* = 0.19. These results indicate that our sample size is statistically sufficient for our primary analyses.

### Statistical analysis

Analyses were conducted using R (4.4.2, The R foundation for statistical computing). Descriptive statistics were computed for statistical analysis. Unless stated otherwise, continuous or ordinal variables are expressed as medians with interquartile ranges [IQRs]. The mean (standard deviation) was used if a normal distribution was visually confirmed. Categorical variables were reported as frequencies, percentages, and 95% confidence interval (95% CI). Fisher's exact test was used to compare categorical variables, whereas the Kruskal–Wallis rank test was used to compare continuous or ordinal variables when data were independent. One-way analysis of variance was used to compare independent parametric variables between three groups. McNamar’s test was used to compare categorical variables between two groups, while the Wilcoxon signed-rank test was used to compare two groups with nonparametric variables.

Before statistical analysis, the dataset obtained from the SMAP–HoPe study and the present study were merged using research IDs. Data from ICUs that were not included in the present study were excluded. Subsequently, missing values were identified. For missing values in the HADS and IES-R, the "half rule" method was used for imputation, whereby the mean of available responses was substituted when at least 50% of the items within a subscale had been completed [20]. Other missing variables other than the outcome were imputed using multiple imputation by chained equations (MICE) before multivariable analysis. In total, 20 imputed datasets were generated (*m* = 20), with 50 iterations performed for each imputation to ensure convergence and stability of the results.

To assess selection bias, we compared the inpatient characteristics, events, and mental health data of respondents and nonrespondents from the SMAP–HoPe study. Representative values for anxiety, depression, and PTSD symptom scores were presented alongside the proportions of individuals exceeding the cutoff thresholds for each condition, with corresponding 95% CIs at both 1 and 4 years post-ICU discharge. For visualization, box plots and individual plots were used to illustrate HADS-A, HADS-D, and IES-R scores at 1 and 4 years post-ICU discharge. Data from the same patients were connected with lines to indicate changes over time. The Wilcoxon test was used to compare each variable between these time points.

In figures and tables illustrating longitudinal changes, only patients with available data at 1- and 4-year follow-ups were included. Therefore, the number and characteristics of participants may differ slightly from the total sample described elsewhere in the manuscript.

Furthermore, the prevalence of anxiety, depression, and PTSD symptoms at 1 and 4 years post-discharge was calculated and presented using a forest plot. Venn diagrams were created using data from 4 years post-discharge with the *eulerr* package in R (version 7.0.2). To examine the relationships between anxiety, depression, and PTSD symptoms, we conducted correlation analyses using Pearson's correlation coefficients. Specifically, we analyzed: (1) correlations between HADS-A, HADS-D, and IES-R scores at 1 year post-ICU discharge; (2) correlations between these scores at 4 years post-ICU discharge; and (3) correlations between the changes in scores (4-year minus 1-year scores) for each domain. Statistical significance was set at p < 0.05 for all correlation analyses.

We used growth mixture models (GMM) to identify latent subgroups of growth change patterns in longitudinal data [21]. We conducted analyses using the *mclust* package. GMM was implemented for three scales—HADS-A, HADS-D, and IES-R. To determine the optimal number of classes, we estimated models with 1–5 classes for each scale and evaluated them based on the Bayesian Information Criterion (BIC), entropy, and substantive interpretability. Lower BIC and entropy values closer to 1 were considered preferable. Additionally, we considered that each class should include at least 5% of the total sample and be theoretically interpretable.

Class-specific mean change patterns and their corresponding 95% CIs for each symptom measure were calculated using standard errors derived from the model estimations. We removed missing data on mental status. The change pattern visualizations included the class-specific mean change pattern with 95% CIs and individual change patterns. Separate GMM analyses were performed for depression, anxiety, and PTSD symptoms, and change pattern plots were created to display class-specific mean and individual change patterns.

GMM was applied to each outcome (HADS-A, HADS-D, and IES-R), identifying three latent classes based on symptom severity at the 1-year follow-up. For the HADS-A and HADS-D, severity levels were defined as follows: scores < 4 were categorized as “minimal,” scores ≥ 4 as “mild,” and scores ≥ 8 as “moderate.” For the IES-R, scores < 4 were categorized as “minimal,” scores ≥ 4 as “mild,” and scores ≥ 10 as “moderate.” These cutoffs were approximately based on quartile distributions. In addition, each class was further labeled according to the symptom change patterns over the 4-year period as “increasing,” “decreasing,” or “stable.” Thus, each class was defined based on baseline symptom severity and its longitudinal change pattern over time.

Univariate analyses were used to clarify the differences in characteristics between classes for each aspect of mental health. Since the comparisons involved more than three groups, the Kruskal–Wallis or Fisher’s exact test was used to determine whether each characteristic was associated with class differences.

In addition, we conducted multivariable analyses to explore factors associated with each class in the three mental health disorders. Variables with a *p* value < 0.1 in the univariate analysis were included in the multinomial logistic regression model. Age was included in all multivariate analyses as a clinically important variable regardless of its statistical significance. We chose *p* < 0.1 as our selection threshold to include factors that might be clinically important, even if they were not statistically significant (*p* < 0.05). The multinomial logistic regression was performed using the “*nnet*" package in R. Class 1 was set as the reference group, and adjusted odds ratios (aORs), 95% CIs, and p-values were calculated. All analyses employed two-sided and statistical significance set at *p* < 0.05.

### Sensitivity analysis

To assess the robustness of the findings derived from the MICE-imputed datasets, we conducted a sensitivity analysis using available case analysis. In this approach, we used only cases with complete data using the variables required for each specific analysis, which resulted in varying sample sizes across the analyses. This sensitivity analysis enabled the evaluation of the impact of the imputation method on the study findings.

## Results

### Recruitment flow chart

Additional file 2 shows a flowchart of the recruitment process. Of the 12 institutions in the previous survey [6], 7 were included in this study. From these 7 institutions, the original group comprised 414 participants. After excluding 95 patients based on the established criteria, 319 participants qualified for the postal survey. Of them, 223 respondents completed the survey, resulting in a response rate of 70.0%. However, only a small number of these postcards were returned, and we were unable to confirm eligibility status in most cases. Consequently, some individuals who were deceased or otherwise ineligible may have been classified as nonresponders.

### Missing values

The characteristics of the patients and missing data on mental health outcomes 1 year post-discharge were fully imputable. In contrast, in the present dataset from the 4-year follow-up, the HADS-A (*n* = 5), HADS-D (*n* = 10), and IES-R (*n* = 23) scores were required. Attempts were made to impute these missing values using the “half rule.” However, it was impossible to impute values for five cases of HADS-A, three cases of HADS-D, and eight cases of IES-R. The details of missing data are presented in Additional file 3.

### Characteristics between responders and nonresponders

We compared in-hospital characteristics, mental health status at 1-year post-discharge, and QOL utility scores derived from the EQ-5D-5L between respondents and nonrespondents (Additional file 4). Among the respondents, 58 (26.0%) were female, which was significantly lower than the 36 (37.5%) among the nonrespondents (*p* = 0.045). The HADS-A scores for respondents (median: 3.50 [1.00–3.50]) were lower than those of nonrespondents (median: 5.00 [2.00–5.00]) (*p* = 0.031). Similarly, HADS-D scores for respondents (mean: 4.67 [2.00–7.00]) were lower than those for nonrespondents (mean: 6.00 [4.00–9.00]) (*p* = 0.01). The QOL utility scores were higher among respondents, with a median score of 0.87 [IQR 0.74–1.00] than 0.81 [IQR 0.65–1.00] for nonrespondents (*p* = 0.04). No statistically significant differences were observed for age, type of admission, APACHE II scores, duration of mechanical ventilation, or ICU length of stay.

### Comparison of mental disorder severity at 1 year versus 4 years after ICU discharge

**Fig. 1:**
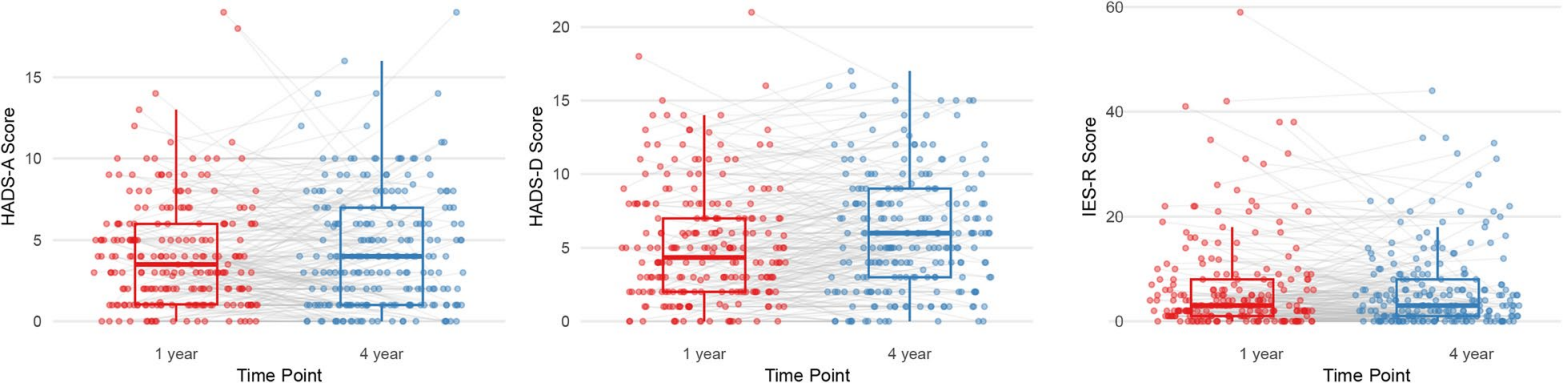
Boxplot for HADS and IES-R scores between 1 and 4 years after ICU discharge

The comparison of HADS-A, HADS-D, and IES-R scores between the 1- and 4-year follow-up periods showed slight changes in these psychological measures over time (Fig. 1). One year following ICU discharge, the HADS-A score was 3.5 [1.1–6.0], while at the 4-year follow-up, the score slightly increased to 4.0 [1.0–7.0] (*p* = 0.201). Similarly, the HADS-D score significantly increased from 4.7 (2.0–7.0) at 1 year to 5.5 (3.0–9.0) after 4 years (*p* < 0.001). In contrast, the IES-R score, used to measure PTSD symptoms, yielded a score of 3.0 [1.0–8.0] at both the 1- and 4-year follow-ups (*p* = 0.135).

Each line represents the change observed in an individual over time. *HADS* Hospital Anxiety and Depression Scale, *IES-R* Impact of Event Scare-Revised, *HADS-A* HADS-anxiety, *HADS-D* HADS-depression

### Comparison of mental disorder prevalence at 1 year versus 4 years after ICU discharge

**Fig. 2:**
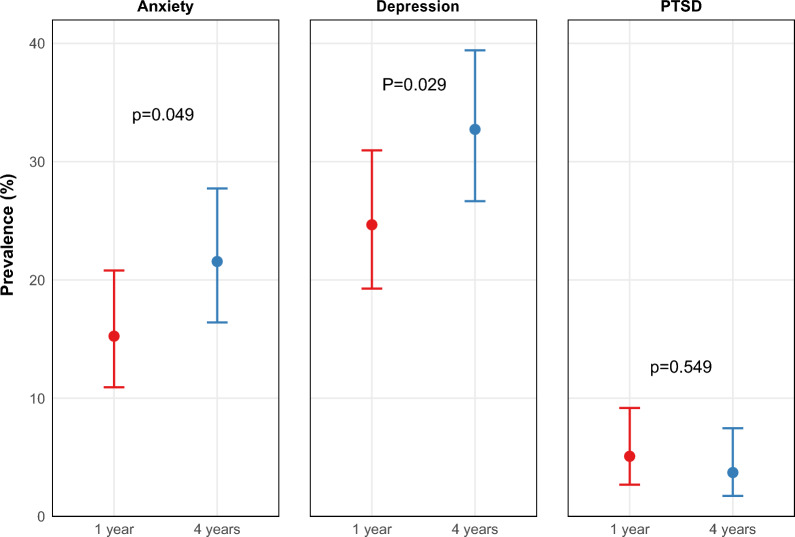
Prevalence of mental health disorders between 1 and 4 years after ICU discharge. The error bar indicates a 95% Confidence Interval. *PTSD* post-traumatic stress disorder

Figure 2 presents a forest plot illustrating the prevalence rates of depression, anxiety, and PTSD symptoms at 1- and 4-years following ICU discharge. The prevalence of anxiety, depression, and PTSD exhibited some variations between the 1- and 4-year periods following ICU discharge. One year after discharge, the prevalence of anxiety symptoms was 15.3% (95% CI: 10.9–20.8), increasing to 21.6% (95% CI: 16.4–27.7) at 4 years (*p* = 0.049). Similarly, the prevalence of depressive symptoms significantly increased from 24.7% (95% CI: 19.3–31.0) at 1 year to 32.7% (95% CI: 26.7–39.4) at 4 years (*p* = 0.021). In contrast, the prevalence of PTSD symptoms slightly decreased from 5.1% (95% CI: 2.7–9.2) at 1 year to 2.7% (95% CI: 1.7– 7.5) at 4 years (*p* = 0.549). The details of the prevalence rate are shown in the Venn diagram in Additional file 5.

### Correlation analysis of symptom changes from 1 to 4 years after ICU discharge

To explore the co-occurrence of changes in anxiety, depression, and PTSD symptoms between 1 and 4 years after ICU discharge, we computed Pearson correlation coefficients among the changed HADS-A, HADS-D, and IES-R scores. Changed scores were defined as differences between scores at the 4-year and 1-year follow-ups. Significant positive correlations were observed between changes in anxiety and depression (*r* = 0.6, *p* < 0.001), depression and PTSD (*r* = 0.34, *p* < 0.001), and anxiety and PTSD (*r* = 0.44, *p* < 0.001). A full matrix of pairwise scatter plots with regression lines, correlation coefficients, and p-values is presented in Additional file 6.

### Change patterns of mental health disorder severity at 1 and 4 years post-ICU discharge

**Fig. 3:**
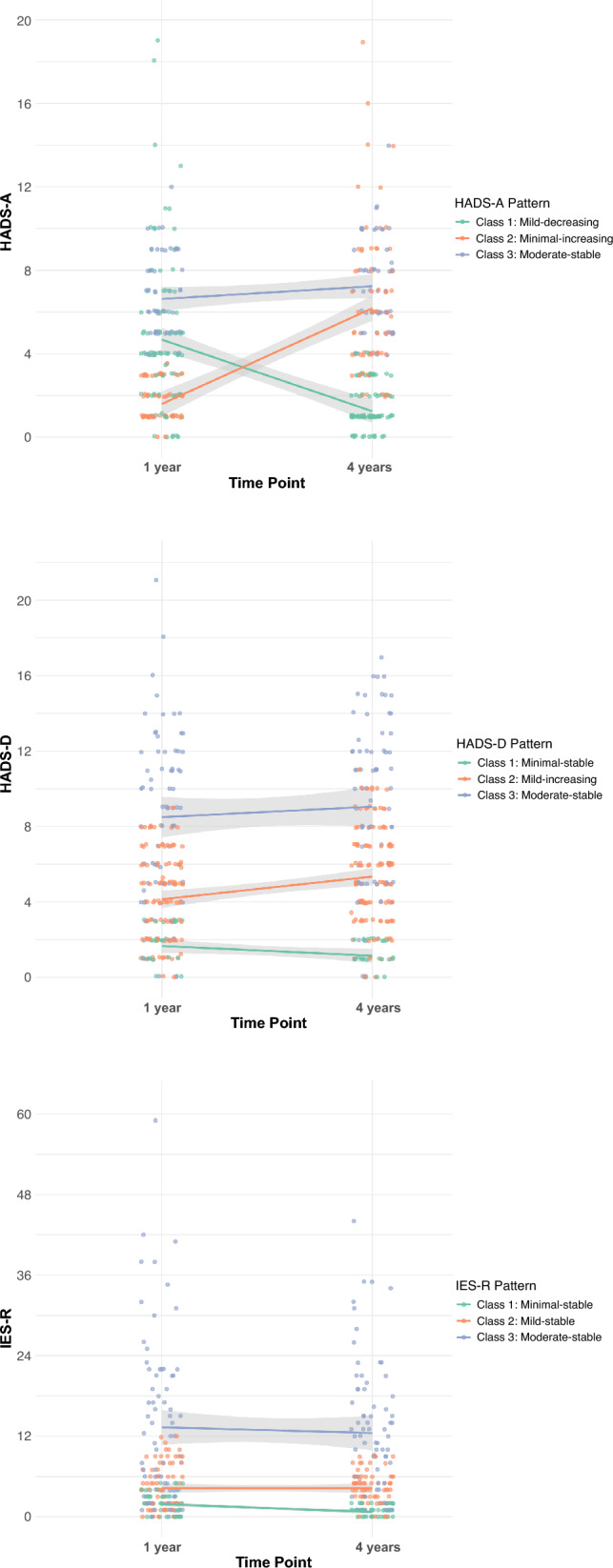
Longitudinal change patterns of three mental health symptom measures identified through growth mixture modeling. Longitudinal change patterns showing the three-class solutions for anxiety (HADS-A, top), depression (HADS-D, middle), and post-traumatic stress (IES-R, bottom). Models show distinct patterns with different stability and change characteristics across the assessment periods. Each dot represents an individual’s score. The gray band represents the 95% confidence interval. *HADS* Hospital Anxiety and Depression Scale, *IES-R* Impact of Event Scale-Revised, *HADS-A* HADS-anxiety score, *HADS-D* HADS-depression score

The results of the GMM are shown in Fig. 3. The details of statistical indices and the GMM process are described in Additional file 7. From the GMM analysis, participants were classified based on the patterns of change in anxiety, depression, and PTSD. For anxiety, three change patterns were identified: “mild-decreasing (class 1, *n* = 91, 42.1%)”; “minimal-increasing (class 2, *n* = 72, 33.3%)”; and “moderate-stable (class 3, *n* = 53, 24.5%).” For depression, participants were grouped into three change patterns: “minimal-stable (class 1, *n* = 27, 12.4%)”; “mild-increasing (class 2, *n* = 121, 55.5%)”; and “moderate-stable (class 3, *n* = 70, 32.1%). “For PTSD, three change patterns were identified: “minimal-stable (class 1, *n* = 59, 28.5%)”; “mild-stable (class 2, *n* = 74, 35.7%)”; “moderate-stable (class 3, *n* = 74, 35.7%)”.

### Univariate analysis of patient characteristics by class

The analysis for anxiety revealed significant differences in the two variables among the three anxiety change patterns (Table 1). While age and sex were similarly distributed across the change pattern classes (*p* = 0.5 and *p* = 0.8, respectively), educational background significantly differed (*p* = 0.002), with higher education being the most prevalent in the mild-decreasing class (39.0%, class 1). Clinical variables, including unemployment status, scheduled surgery, and critical illness severity measures, did not significantly differ between the change pattern classes.

**Table 1:** Comparison of anxiety change patterns and associated factors in patients in the ICU (*N* = 216)

Class	1	2	3	
Predictor variables	Mild-decreasing *n* = 91 (42.1%)	Minimal-increasing *n* = 72 (33.3%)	Moderate-stable *n* = 53 (24.5%)	*p*-value
Age (years), median [IQR]	74.0 [65.0, 81.0]	73.0 [66.5, 83.0]	74.0 [57.0, 80.0]	0.500
Male, n (%)	66 (73.0)	55 (76.0)	38 (72.0)	0.800
Higher education, n (%)	35 (39.0)	14 (20.0)	8 (15.0)	0.002
Unemployed, n (%)	58 (64.0)	51 (72.0)	29 (55.0)	0.140
Scheduled surgery, n (%)	46 (51.0)	36 (50.0)	20 (38.0)	0.300
APACHE II, median [IQR]	15.0 [11.0, 12.0]	15.5 [12.00, 21.00]	13.0 [10.0, 18.0]	0.059
SOFA, median [IQR]	6.50 [4.00, 8.00]	6.00 [4.00, 8.00]	6.00 [5.00, 8.00]	0.643
MV use, n (%)	24 (75.0)	70 (81.4)	81 (84.4)	0.486
Days of MV use, median [IQR]	2.0 [1.0, 3.0]	2.0 [2.0, 4.0]	2.0 [1.0, 4.0]	0.130
Days of delirium, median [IQR]	0.0 [0.0, 1.0]	1.0 [0.0, 2.0]	0.0 [0.0, 1.0]	0.378
Days of coma, median [IQR]	0.0 [0.0, 1.0]	1.0 [0.0, 1.0]	0.0 [0.0. 1.0]	0.400
ICU LOS (days), median [IQR]	5.0 [4.0, 7.0]	5.5 [4.0, 7.5]	6.0 [4.0, 7.0]	0.500
Hospital LOS (days), median [IQR]	29.0 [21.0, 45.0]	25.0 [21.0, 38.0]	24.0 [16.0, 35.0]	0.110
Malignancy (%)	10 (11)	7 (9.7)	9 (17)	0.400

*IQR* interquartile range, *APACHE II* Acute Physiology and Chronic Health Evaluation II, *SOFA* Sequential Organ Failure Assessment, *MV* mechanical ventilation, *ICU* intensive care unit, *LOS* length of stay

The analysis for depression revealed three distinct change patterns (Table 2). Educational background was the only significant differentiating factor (*p* = 0.037), with higher education being the most common in the minimal-stable (Class 1) class (42.0%). Demographic and clinical characteristics, including age, sex, illness severity, and length of stay, did not significantly differ among the change pattern classes.

**Table 2:** Comparison of depression change patterns and associated factors in patients in the ICU (*N* = 218)

Class	1	2	3	
Predictor variables	Minimal-stable *n* = 27 (12.4%)	Mild-increasing *n* = 121 (55.5%)	Moderate-stable *n* = 70 (32.1%)	*p*-value
Age (years), median [IQR]	74.0 [67.0, 77.0]	74.0 [65.0, 83.0]	73.5 [60.0, 81.0]	0.800
Male, n (%)	20 (74.0)	94 (78.0)	47 (67.0)	0.300
Higher education, n (%)	11 (42.0)	35 (29.0)	12 (17.0)	0.037
Unemployed, n (%)	19 (73.0)	71 (59.0)	50 (71.0)	0.150
Scheduled surgery, n (%)	13 (48.0)	61 (50.0)	30 (43.0)	0.600
APACHE II, median [IQR]	14.0 [10.0, 19.0]	15.0 [12.0, 20.0]	14.0 [11.0, 19.0]	0.400
SOFA, median [IQR]	6.00 [4.00, 8.00]	7.00 [5.00, 9.00]	6.00 [4.75, 8.00]	0.358
MV use, n (%)	40 (80.0)	40 (74.1)	100 (86.2)	0.150
Days of MV use, median [IQR]	2.0 [0.0, 3.0]	2.0 [2.0, 4.0]	2.0 [0.0, 3.0]	0.093
Days of delirium, median [IQR]	0.0 [0.0, 1.0]	0.0 [0.0, 1.0]	0.0 [0.0, 2.0]	0.500
Days of coma, median [IQR]	1.0 [0.0, 1.0]	0.0 [0.0, 1.0]	0.0 [0.0. 1.0]	0.400
ICU LOS (days), median [IQR]	5.0 [4.0, 6.0]	6.0 [4.0, 8.0]	5.0 [4.0, 7.0]	0.140
Hospital LOS (days), median [IQR]	29.0 [22.0, 36.0]	26.0 [20.0, 41.0]	25.0 [16.0, 37.0]	0.400
Malignancy (%)	4 (15)	13 (11)	10 (14)	0.700

*IQR* interquartile range, *APACHE II* Acute Physiology and Chronic Health Evaluation II, *SOFA* Sequential Organ Failure Assessment, *MV* mechanical ventilation, *ICU* intensive care unit, *LOS* length of stay

Regarding PTSD, we identified three change patterns with nearly equal distribution (Table 3). Three factors significantly differentiated these change patterns: higher education (*p* = 0.004) had the highest prevalence in the mild-stable class (39.0%, class 2); scheduled surgery (*p* = 0.041) was most common in the mild-stable class (55.0%, class 2); and days of delirium (*p* = 0.036) was more frequent in the moderate-stable class (class 3) showed slightly higher delirium duration. Age, sex, unemployment status, illness severity measures, and length of stay did not significantly differ among the change pattern classes.

**Table 3:** Comparison of post-traumatic stress disorder growth change patterns and associated factors in patients in the ICU (N = 207)

Class	1	2	3	
Predictor variables	Minimal-stable *n* = 59 (28.5%)	Mild-stable *n* = 74 (35.7%)	Moderate-stable *n* = 74 (35.7%)	*p*-value
Age (years), median [IQR]	74.0 [67.0, 82.0]	72.5 [63.0, 80.0]	73.0 [63.0, 81.0]	0.800
Male, n (%)	50 (85.0)	51 (69.0)	50 (68.0)	0.054
Higher education, n (%)	15 (26.0)	29 (39.0)	11 (15.0)	0.004
Unemployed, n (%)	35 (60.0)	46 (62.0)	48 (65.0)	0.900
Scheduled surgery, n (%)	29 (49.0)	41 (55.0)	26 (35.0)	0.041
APACHE II, median [IQR]	14.0 [11.0, 20.0]	13.5 [10.0, 19.0]	15.0 [13.0, 20.0]	0.200
SOFA, median [IQR]	6.00 [5.00, 8.00]	6.00 [4.00, 7.00]	6.00 [5.00, 9.00]	0.753
MV use, n (%)	61 (82.4)	33 (78.6)	26 (83.9)	0.923
Days of MV use, median [IQR]	2.0 [1.0, 3.0]	2.0 [1.0, 3.0]	2.0 [1.0, 5.0]	0.700
Days of delirium, median [IQR]	0.0 [0.0, 1.0]	0.0 [0.0, 1.0]	1.0 [0.0, 2.0]	0.036
Days of coma, median [IQR]	1.0 [0.0, 1.0]	0.0 [0.0, 1.0]	0.0 [0.0. 2.0]	0.200
ICU LOS (days), median [IQR]	5.0 [4.0, 6.0]	6.0 [4.0, 7.0]	6.0 [4.0, 8.0]	0.200
Hospital LOS (days), median [IQR]	30.0 [21.0, 42.0]	28.0 [20.0, 35.0]	25.0 [17.0, 40.0]	0.600
Malignancy (%)	8 (14)	5 (6.8)	13 (18)	0.130

*IQR* interquartile range, *APACHE II* Acute Physiology and Chronic Health Evaluation II, *SOFA* Sequential Organ Failure Assessment, *MV* mechanical ventilation, *ICU* intensive care unit, *LOS* length of stay

### Multivariable analysis to identify factors for each class

Owing to the presence of missing data in the dataset, MICE was employed before the multivariable analysis.

Table 4 presents the results of the multinomial logistic regression for the anxiety change pattern classes, with the mild-decreasing (Class 1) group serving as the reference. When the reference was compared with the minimal-increasing (Class 2) class, higher education emerged as a significant predictor (aOR = 0.40, 95% CI: 0.19–0.85, *p* = 0.016). This predictive effect was even stronger when the reference was compared with the moderate-stable (Class 3) class, where higher education was associated with significantly lower odds of being in this class (aOR = 0.24, 95% CI: 0.10–0.59, *p* = 0.002). Age, days of delirium, and APACHE II scores were not significantly associated with the anxiety change pattern classes.

**Table 4:** Multinomial logistic regression analysis of factors associated with anxiety change pattern classes assessed using multinomial logistic regression analysis

	Mild-decreasing (Class 1) vs. Minimal-increasing (Class 2)		Mild-decreasing (Class 1) vs. Moderate-stable (Class 3)	
	aOR (95% CI)	*p*-value	aOR (95% CI)	*p*-value
Age	1.01 (0.98, 1.03)	0.595	0.99 (0.96, 1.02)	0.457
Higher education	0.40 (0.19, 0.85)	0.016	0.24 (0.10, 0.59)	0.002
Days of delirium	1.05 (0.89, 1.26)	0.600	0.93 (0.73, 1.19)	0.550
APACHE II	1.02 (0.96, 1.07)	0.561	0.95 (0.89, 1.01)	0.108

Mild-decreasing (Class 1) indicates reference class

*APACHE II* Acute Physiology and Chronic Health Evaluation II, *aOR* adjusted odds ratio, *CI* confidence interval

Table 5 presents the multinomial logistic regression results for the depression change pattern classes, with the minimal-stable (Class 1) class serving as the reference. When the reference was compared with the mild-increasing (Class 2) class, none of the examined variables emerged as significant predictors, including higher education (aOR = 0.56, 95% CI: 0.23–1.35, *p* = 0.194), age (aOR = 0.99, 95% CI: 0.95–1.03, *p* = 0.512), and days of mechanical ventilation use (aOR = 1.18, 95% CI: 0.94–1.48, *p* = 0.140). However, when the reference was compared with the moderate-stable (Class 3) class, higher education was a significant predictor (aOR = 0.25, 95% CI: 0.09–0.68, *p* = 0.006), associated with lower odds of being in the moderate-stable depression class. Age and days of mechanical ventilation were not significantly associated with the depression change pattern classes.

**Table 5:** Factors associated with depression change pattern classes assessed using multinomial logistic regression analysis

	Minimal-stable (Class 1) vs. Mild-increasing (Class 2)		Minimal-stable (Class 1) vs. Moderate-stable (Class 3)	
	aOR (95% CI)	*p*-value	aOR (95% CI)	*p*-value
Age	0.99 (0.95, 1.03)	0.512	0.98 (0.94, 1.02)	0.263
Higher education	0.56 (0.23, 1.35)	0.194	0.25 (0.09, 0.68)	0.006
Days of MV use	1.18 (0.94, 1.48)	0.140	1.07 (0.85, 1.35)	0.567

Minimal-stable (Class 1) indicates reference class

*MV* mechanical ventilation, *aOR* adjusted odds ratio, *CI* confidence interval

Table 6 presents the results of the multinomial logistic regression for the PTSD change pattern classes. Class 1 (minimal-stable) served as the reference group. Notably, when it was compared with the mild-stable (Class 2) class, two variables emerged as significant predictors—male sex (aOR = 0.32, 95% CI: 0.13–0.79, *p* = 0.013) was associated with lower odds of being in the mild-stable class (Class 2), whereas higher education (aOR = 2.28, 95% CI: 1.03–5.02, *p* = 0.041) was associated with increased odds of mild-stable (Class 2) change patterns. In the comparison between the reference and moderate-stable (Class 3) class, no variable was statistically significant, although male sex showed a slight significance (aOR = 0.42, 95% CI: 0.17–1.02, *p* = 0.054). Age, scheduled surgery, and days of delirium were not significantly associated with the PTSD change pattern class in either comparison.

**Table 6:** Factors associated with post-traumatic stress disorder change pattern classes assessed using multinomial logistic regression analysis

	Minimal-stable (Class 1) vs Mild stable (Class 2)		Minimal-stable (Class 1) vs Moderate-stable (Class 3)	
	aOR (95% CI)	*p*-value	aOR (95% CI)	*p*-value
Age	0.99 (0.96, 1.02)	0.425	0.98 (0.96, 1.01)	0.277
Male	0.32 (0.13, 0.79)	0.013	0.42 (0.17, 1.02)	0.054
Higher education	2.28 (1.03, 5.02)	0.041	0.62 (0.25, 1.51)	0.291
Scheduled surgery	1.47 (0.70, 3.07)	0.306	0.73 (0.35, 1.54)	0.411
Days of delirium	1.16 (0.88, 1.52)	0.291	1.25 (0.97, 1.60)	0.089

Minimal-stable (Class 1) indicate reference class

*aOR* adjusted odds ratio, *CI* confidence interval

### Sensitivity analysis

To evaluate the robustness of our findings, we performed a sensitivity analysis comparing results from the primary analysis using MICE-imputed data with those from available case analyses (Additional file 8). The patterns of associations between predictors and psychological outcomes were mainly consistent across both analytical approaches.

## Discussion

In this study, we evaluated the 4-year course of mental health disorders after ICU discharge and compared it with mental health disorders at 1 year. The main result showed that the prevalence of depression significantly increased from 24.7% at 1 year to 32.7% at 4 years, and anxiety showed an increasing trend from 15.3% to 21.6%, while the prevalence of PTSD slightly decreased from 5.1% to 2.7%. Furthermore, analysis of the change pattern of each mental health disorder revealed multiple change patterns for anxiety, depression, and PTSD, and higher education was associated with anxiety and depression mental health change patterns.

The long-term persistence of mental health disorders, particularly for 4 years after ICU discharge, with the increase in depression prevalence, indicates that PICS-related mental health problems do not naturally improve over time. This finding is consistent with those of previous studies, which have shown long-term persistence of mental health problems in ICU survivors, including a 5-year longitudinal study involving patients with acute respiratory distress syndrome (ARDS) [7].

A key finding of this study is the significant increase in the prevalence of depression over time. This contrasts with the findings of a previous 5-year longitudinal study including patients with ARDS, which reported that mental health symptoms such as anxiety and depression showed little evidence of sustained improvement and tended to persist or recur in many patients [7]. However, the study also found no consistent trend toward worsening symptoms during the follow-up period. One possible reason for this discrepancy is the differences in patient populations. Prior studies often included younger individuals with a narrower range of diagnoses; for example, the median age in the ARDS study was 49 years, which is considerably lower than the median age of our cohort [7]. Age-related factors may play an important role in the worsening of depressive symptoms, as consistently reported in both community-dwelling older adult populations and individuals with chronic illnesses such as cancer [22, 23].

In addition, population-based studies using matched cohorts have demonstrated a higher cumulative incidence of newly diagnosed mental health disorders, such as depression and anxiety, in ICU survivors than in matched controls from the general population [24]. These studies typically relied on diagnostic codes recorded in administrative databases. While useful for estimating the emergence of new diagnoses at a population level, they do not help assess symptom resolution or the overall prevalence. In particular, they cannot determine whether depressive symptoms have persisted, improved, or worsened over time in individual patients. Therefore, these studies provide a limited and potentially biased view of post-ICU mental health trajectories.

Furthermore, our study followed the same patient cohort longitudinally over 4 years using validated psychological assessment tools. This design enabled us to capture not only the emergence of new symptoms but also the actual progression—either worsening or improvement—of the existing symptoms. This nuanced perspective offers a more comprehensive understanding of how mental health evolves in ICU survivors and highlights the need for long-term monitoring that extends beyond simple diagnostic incidence.

Several factors may explain the increase in depression despite the alleviation of PTSD symptoms. Notably, our follow-up period (2022–2023) overlapped with the COVID-19 pandemic, which may have contributed to this trend. The pandemic introduced chronic stressors including prolonged social isolation, restrictions on daily activities, limited healthcare access, and economic instability, which are known to be strongly associated with the development and worsening of depression than acute traumatic experiences [27]. These factors may have exacerbated underlying vulnerabilities in ICU survivors. Additionally, while PTSD is generally linked to specific traumatic memories that may fade over time, depression tends to be more closely related to ongoing functional limitations and cumulative life stressors [27, 28].

Our findings showed that depression and anxiety exhibited an increasing trend; however, this trend may not be unique to post-ICU patients. As we previously noted, studies have shown that advancing age is associated with high rates of depression in non-ICU population [22–24]. A study identified older age as a risk factor for depression in patients with cancer [22]. Additionally, population-based study has reported that the prevalence of depression increases with age, especially among individuals aged 76 years and older, with statistically significant trends [23]. These findings are consistent with our results. Nevertheless, the multivariate analysis in the present study revealed that age was not related to the prevalence of depression. This may be due to the sampling bias, as the study population primarily consisted of older adults, potentially limiting statistical power.

Additionally, one plausible explanation for the increased prevalence of depression among older adults is the deterioration of physical function. In post-ICU patients, grip strength has been shown to correlate negatively with HADS scores [25], suggesting a link between physical and psychological recovery. Moreover, previous studies have demonstrated that physical decline is associated with increased depressive severity [26]. ICU survivors often experience a dual burden—age-related physiological decline and ICU-acquired physical impairments. Therefore, the observed increase in depression over time should be interpreted with respect to ICU-specific sequelae and aging-related factors. While this trend is not exclusive to ICU survivors, the intersection of critical illness and normal aging may render this population particularly susceptible to long-term psychological deterioration.

In this study, changes in anxiety, depression, and PTSD symptoms over time demonstrated significant correlation, suggesting that psychological symptom change after ICU discharge are, to some extent, interrelated rather than independent. This finding is consistent with previous findings revealing that emotional symptoms in ICU survivors often co-occur and fluctuate together over time [29]. However, we also observed that the correlation between changes in PTSD symptoms and changes in anxiety or depression was somewhat weaker than the correlation between anxiety and depression. This finding suggests that PTSD symptoms may share some, but not all, underlying mechanisms with anxiety and depression. PTSD is characterized by re-experiencing, avoidance, and hyperarousal, which may be more closely linked to ICU-specific traumatic experiences such as pain or physical restraint during mechanical ventilation [30, 31]. These distinctions may partly explain the weaker correlations observed and highlight the heterogeneous nature of psychological responses following critical illness.

The different change patterns identified from the GMM analysis showed that mental health problems in ICU survivors are not uniform, and the course differs among individuals. This is a major finding revealing that while some patients remain stable with mild symptoms, others experience moderate to severe symptoms that persist or worsen. This finding is consistent with those of Schmidt et al.’s study [8] on PTSD trajectories and Boede's study [9] on depression trajectories, both in patients with sepsis. Their findings showed diverse trajectories regarding mental health severity, indicating that mental health progression after a patient’s discharge from the ICU is not "uniform," but individual change patterns exist. However, direct comparison is challenging due to methodological differences. Schmidt et al. [8] studied sepsis survivors (median age, 61 years) over 2 years in Germany, while Boede et al. examined sepsis patients (mean age, 61.4 years) over 12 months. In contrast, our study included broader ICU populations, older patients, and 4-year follow-up in Japan.

Educational history may influence post-discharge mental health trajectories over 1–4 years. In this study, higher education was associated with a lower likelihood of worsening or severe anxiety and depression symptoms. This finding is consistent with those of previous reports indicating that individuals with higher education levels are less likely to experience mental health disorders. For instance, a study involving 2,345 ICU survivors found that higher education was protective against any mental health condition [32], and other studies have shown that higher education is linked to reduced anxiety and depression symptoms [33, 34].

The mechanisms underlying this association are not fully understood. However, a past review has suggested that higher education may enhance a sense of personal control [35] and improve health literacy, thereby facilitating access to health information and promoting healthier behaviors. A structural equation modeling study among Japanese adults further indicated that self-rated health is indirectly influenced by health literacy, which itself is shaped by educational background [36]. Notably, socioeconomic status (SES) has been associated with worse mental health outcomes [37, 38], and SES itself is often correlated with educational attainment [39]. Therefore, educational history may serve as a proxy for SES in this context, and this should be taken into account when interpreting the observed associations.

Our analysis also revealed an unexpected finding regarding PTSD symptom change pattern that higher education was associated with greater odds of belonging to the mild-stable group than the low-stable group (aOR = 2.28, 95% CI: 1.03–5.02, *p* = 0.041). However, this finding should be interpreted with caution. Both groups displayed PTSD symptom levels substantially below the diagnostic threshold, and the absolute difference in severity between the groups was small. Thus, although statistically significant, the clinical relevance of this difference may be limited. Furthermore, the lower bound of the CI was close to 1.0, indicating marginal statistical significance and some uncertainty in the result.

In addition to education, male sex was significantly associated with PTSD change patterns. Some clinical variables, such as delirium duration, showed trends toward significance in univariate or multivariable models, but these did not reach statistical significance—possibly due to limited sample size.

Our findings showed no significant association between acute illness severity or ICU treatment duration and long-term mental health outcomes. This finding aligns with those of previous research and meta-analysis research indicating that commonly used severity scores (e.g., APACHE II, SOFA) and ICU length of stay are not reliable predictors of depression, anxiety, or PTSD after ICU discharge [6, 40]. Previous study reported that 1-year physical and mental health problems in ICU survivors were more strongly associated with pre-ICU health status and sociodemographic factors than with severity of illness or ICU length of stay [32]. In this context, long-term mental health appears to be less determined by the intensity of the initial illness and more by the broader recovery environment and individual psychosocial factors.

Our findings underscore the importance of sustained follow-up care for ICU survivors, especially considering the observed worsening of depressive symptoms over time. Long-term follow-up may help identify patients at risk of psychological decline and enable timely support. Despite growing awareness of post-ICU impairments, structured follow-up services remain underutilized worldwide, with notable international differences. For instance, only 3.6% of ICUs in Japan and 2% in Australia offer such services [41, 42]. These findings reflect a gap between the need for long-term care and its practical implementation. In addition, certain populations, such as, such as individuals with lower educational attainment, may require special consideration when designing follow-up strategies. The observed association between higher education and lower risk of mental health deterioration may indicate that individuals with lower educational attainment could benefit from a more tailored and continuous support. During ICU care, clear communication about psychological risks and early signs of vulnerability may be helpful. Following discharge, simplified educational materials, family involvement, and screening may assist in identifying those at risk. These approaches may need to be adjusted based on individual backgrounds and health literacy [43, 44]. While our findings highlight the value of accessible and structured mental health follow-up services, these implications should be interpreted with caution due to the observational nature of the study.

This study has some limitations. First, baseline characteristics differed significantly between responders and nonresponders. Specifically, nonresponders had higher levels of anxiety and depression symptoms and lower QOL utility scores at baseline than responders. These differences suggested that individuals with poorer psychological health and quality of life were less likely to participate in follow-up assessments. Consequently, our findings may underestimate the actual prevalence and severity of long-term mental health problems among ICU survivors. Furthermore, because these individuals may have been more likely to follow a worsening or persistently severe symptom change pattern, the proportion of patients in higher-risk change pattern classes may also be underestimated. This potential nonresponse bias limits the generalizability of our findings and should be considered when interpreting the overall patterns of mental health outcomes reported in this study. Second, the potential misclassification of some nonresponders in another limitation. Although we sent confirmation letters before distributing the follow-up questionnaires to assess exclusion criteria, not all individuals meeting those criteria responded. Thus, the nonresponder group may have included patients who had died or were unable to complete the questionnaire due to severe disability or cognitive impairment. Third, mental health assessments were based on self-reported questionnaires rather than clinical diagnoses by psychiatrists. While these scales are widely used in many studies [45], they have limitations in diagnostic accuracy. Fourth, the lack of intermediate assessments between the 1- and 4-year time points prevents a detailed understanding of whether changes in mental health status were linear or nonlinear. Considering the long-term follow-up, events that occurred during this period may have influenced the results. The lack of data on major life events during the follow-up period limits our ability to determine whether observed changes in mental health reflect natural progression or responses to specific stressors. This is particularly important in older ICU survivors, who are more likely to experience major events such as illness, bereavement, or social and economic changes.　To address this limitation, future studies should incorporate more frequent assessments (e.g., annually) and systematically collect data on life events during follow-up. Such event-sensitive designs would enable a clearer understanding of the mechanisms underlying mental health changes and better capture long-term psychological outcomes in aging ICU populations.　Fifth, in comparisons involving small latent classes, the limited sample size may result in insufficient statistical power. Consequently, the absence of statistically significant findings does not necessarily imply the absence of a true association due to the potential for type II errors. Moreover, in such small groups, the estimated effect sizes and CIs tend to be unstable, requiring cautious interpretation. Sixth, although the follow-up was conducted in only 7 of the 12 ICUs from the original study [6], primarily due to logistical constraints, we cannot rule out the possibility of selection bias. The impact of nonparticipation on the results remains uncertain. Seventh, in this study, days with both delirium and coma were counted only as delirium days. It may have underestimated the total duration of coma or failed to capture the complexity of overlapping neurological states. Finally, the follow-up period of this study (2022–2023) overlapped with the COVID-19 pandemic, which may have influenced participants’ mental health independent of their ICU experience. Although we did not collect pandemic-specific data, the potential confounding effects of these external stressors should be considered when interpreting our findings.

The study contributes to the existing body of research by identifying distinct change patterns in long-term mental health disorders among ICU survivors and elucidating factors associated with these patterns. In particular, the finding that higher educational attainment may be protective against more severe mental health change patterns, and that sex appears to influence PTSD change patterns, offers valuable insight for the development of more tailored prevention and intervention strategies.

## Conclusions

This observational study suggests that depression may increase over time among ICU survivors, whereas PTSD symptoms may slightly decrease. Distinct change patterns were identified, with higher education associated with more favorable change patterns in depression and anxiety. Although causality cannot be inferred, these findings support the possible need for long-term psychological follow-up. A feasible clinical framework may include periodic mental health screening, multidisciplinary post-ICU follow-up, and patient education tailored to individual needs.

## Supplementary Information

Additional file 1. Descriptions and scoring criteria for the psychological assessment scales (HADS and IES-R) used in this study.

Additional file 2. Participants’ recruitment flow chart.

Additional file 3. Number of missing items.

Additional file 4. Demographics of respondents and nonrespondents.

Additional file 5. Venn diagram for anxiety, depression, and post-traumatic stress disorder in patients 4 years after ICU discharge.

Additional file 6. Correlation matrix of symptom changes in anxiety, depression, and PTSD from 1-year to 4-year follow-up.

Additional file 7. Statistical indices for evaluating growth mixture models in longitudinal analysis of psychological symptom change patterns.

Additional file 8. Sensitivity analysis using available case analysis: factors associated with anxiety, depression, and post-traumatic stress disorder change pattern classes.

## Data Availability

The datasets used and/or analyzed during the current study are available from the corresponding author on reasonable request.

## Abbreviations

ICU: Intensive care unit
PICS: Post-intensive care syndrome
HADS: Hospital Anxiety and Depression Scale
HADS-A: Hospital Anxiety and Depression Scale Anxiety subscale
HADS-D: Hospital Anxiety and Depression Scale Depression subscale
IES-R: Impact of event scale-revised
PTSD: Post-traumatic stress disorder
APACHE II: Acute Physiology and Chronic Health Evaluation II
SOFA: Sequential Organ Failure Assessment
RASS: Richmond Agitation-Sedation Scale
EQ-5D-5L: EuroQol 5 dimension 5 level
MV: Mechanical ventilation
GMM: Growth mixture model
aOR: Adjusted odds ratio
CI: Confidence interval
LOS: Length of stay
QOL: Quality of life
