# Supplementary material for: Long-term mental health change patterns in ICU survivors: a four-year comparative follow-up from the SMAP–HoPe study
Source: J Intensive Care. 2025 Jul 28;13:41. doi: 10.1186/s40560-025-00812-z (PMC12302793; doi:10.1186/s40560-025-00812-z)
Supplement: Supplementary file 1 — Additional file 1. Descriptions and scoring criteria for the psychological assessment scales (HADS and IES-R) used in this study. [file 40560_2025_812_MOESM1_ESM.docx]

**Additional file 1**

**Descriptions and Scoring Criteria for the Psychological Assessment Scales (HADS and IES-R) Used in This Study**

The impact of event score (IES-R), a widely used instrument for evaluating post-traumatic stress disorder (PTSD), comprises 22 items that are used to measure the level of distress experienced in the past week, with each item rated on a scale from 0 (not at all) to 4 (extremely). It includes three subscales: intrusion, avoidance, and hyperarousal. The Japanese version of the IES-R has sensitivity and specificity ranges of 0.75–0.89 and 0.71–0.93, respectively, using a cutoff score of 25 to diagnose partial PTSD [1]. This threshold was used to identify participants exhibiting substantial PTSD-related symptoms. Additionally, HADS is a well-validated and reliable instrument for assessing anxiety and depressive symptoms. It has been widely used in both outpatient settings and critically ill patient populations [2,3]. The Japanese version of the HADS also has strong reliability and validity [4]. The scale comprises 14 items, divided into two subscales: anxiety (HADS-A) and depression (HADS-D). Each item is scored on a scale of 0–3, resulting in subscale scores ranging from 0–21. Previous studies have reported strong correlations between HADS scores and psychiatrist-diagnosed anxiety and depression (Spearman’s correlation: r=0.70 for anxiety and r=0.74 for depression) [4]. In this study, a subscale score of ≥8 was used to indicate clinically significant anxiety or depression, following the criteria established for the Japanese version [4].

1. Asukai N, Kato H, Kawamura N, Kim Y, Yamamoto K, Kishimoto J, et al. Reliability and validity of the Japanese-language version of the impact of event scale-revised (IES-R-J): four studies of different traumatic events. J Nerv Ment Dis. 2002;190:175-82.

2. Zigmond AS, Snaith RP. The hospital anxiety and depression scale. Acta Psychiatr Scand. 1983;67:361-70.

3. Jutte JE, Needham DM, Pfoh ER, Bienvenu OJ. Psychometric evaluation of the Hospital Anxiety and Depression Scale 3 months after acute lung injury. J Crit Care. 2015;30:793-8.

4. Hatta H, Higashi A, Yashiro H, Kotaro O, Hayashi K, Kiyota K, et al. A Validation of the Hospital Anxiety and Depression Scale. Jpn J Psychosom Med. 1998;38:309-15.
