# Supplementary figures and images for: Long-term mental health change patterns in ICU survivors: a four-year comparative follow-up from the SMAP–HoPe study
Source: J Intensive Care. 2025 Jul 28;13:41. doi: 10.1186/s40560-025-00812-z (PMC12302793; doi:10.1186/s40560-025-00812-z)

**Additional file 2**


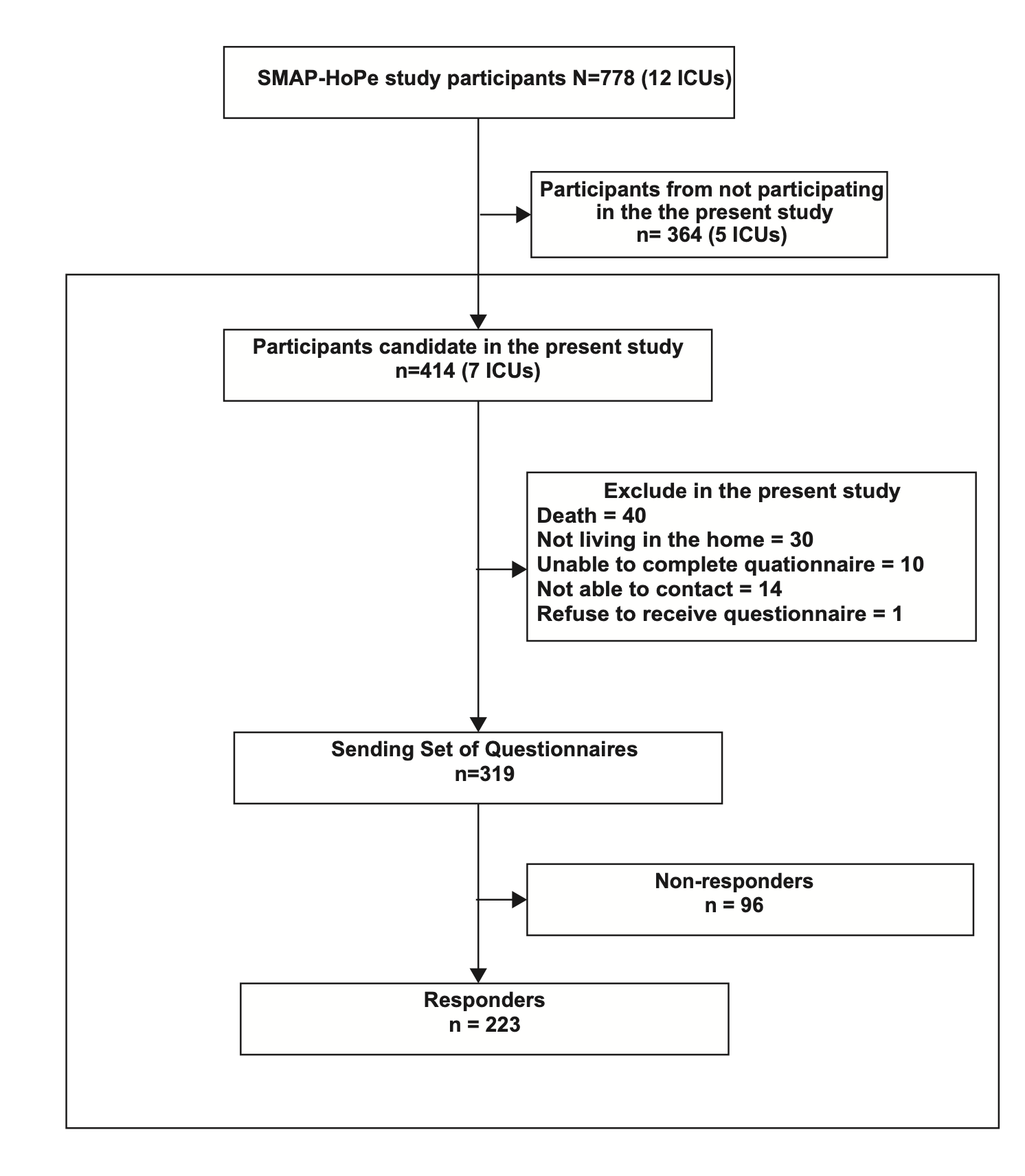


**Participants’ recruitment flow chart**

ICU, intensive care unit.

Supplement: Supplementary file 2 — Additional file 2. Participants’ recruitment flow chart. [file 40560_2025_812_MOESM2_ESM.docx]
