# Supplementary material for: Long-term mental health change patterns in ICU survivors: a four-year comparative follow-up from the SMAP–HoPe study
Source: J Intensive Care. 2025 Jul 28;13:41. doi: 10.1186/s40560-025-00812-z (PMC12302793; doi:10.1186/s40560-025-00812-z)
Supplement: Supplementary file 3 — Additional file 3. Number of missing items. [file 40560_2025_812_MOESM3_ESM.docx]

**Additional file 3**

**Number of missing items**

| **Variables** | **Number of missing (%)** |
| --- | --- |
| Higher education | 7 (3.1) |
| Unemployed | 5 (2.2) |
| HADS anxiety score | 5 (2.2) |
| HADS depression score | 10 (4.5) |
| IES-R score | 23 (10.3) |

HADS, Hospital Anxiety and Depression Scale; IES-R, Impact of Event Scale-Revised.
