# Supplementary material for: Long-term mental health change patterns in ICU survivors: a four-year comparative follow-up from the SMAP–HoPe study
Source: J Intensive Care. 2025 Jul 28;13:41. doi: 10.1186/s40560-025-00812-z (PMC12302793; doi:10.1186/s40560-025-00812-z)
Supplement: Supplementary file 4 — Additional file 4. Demographics of respondents and nonrespondents. [file 40560_2025_812_MOESM4_ESM.docx]

**Additional file 4**

**Demographics of respondents and non-respondents**

| **Variable** | | **Responders (n=223)** | **Non-responders (n=96)** | **p-value** |
| --- | --- | --- | --- | --- |
| Baseline characteristics | | | | |
|  | Age (years), median [IQR] | 73 [64,80] | 74 [65, 82] | 0.475 |
|  | Female sex, n (%) | 58 (26.0) | 36 (37.5) | 0.045 |
|  | Malignancy, n (%) | 28 (12.6) | 11 (11.5) | 0.854 |
|  | History of psychiatric illness, n (%) | 7 (3.1) | 5 (5.2) | 0.356 |
| Type of admission | | | | |
|  | Scheduled surgery, n (%) | 107 (48.0) | 46 (47.9) | 1.000 |
|  | Unscheduled surgery, n (%) | 39 (17.5) | 15 (15.6) | 0.807 |
|  | Medical, n (%) | 77 (33.0) | 35 (36.4) | 0.610 |
| Reason for ICU admission | | | | |
|  | CV surgery, n (%) | 112 (50.2) | 48 (50.0) | 0.514 |
|  | Sepsis, n (%) | 28 (12.6) | 6 (6.2) |  |
|  | CHF / AMI / Arrhythmia, n (%) | 25 (11.2) | 14 (14.6) |  |
|  | ARF, n (%) | 14 (6.3) | 4 (4.2) |  |
|  | Abdominal surgery, n (%) | 9 (4.0) | 3 (3.1) |  |
|  | Aortic dissection (non-operative), n (%) | 9 (4.0) | 5 (5.2) |  |
|  | ENT surgery, n (%) | 4 (1.8) | 2 (2.1) |  |
|  | Trauma, n (%) | 3 (1.3) | 4 (4.2) |  |
|  | Other surgery, n (%) | 10 (4.5) | 3 (3.1) |  |
|  | Others, n (%) | 9 (4.0) | 7 (7.3) |  |
| Severity of illness | | | | |
|  | APACHE II, median [IQR] | 15.00 [11.00, 19.50] | 15.50 [12.00, 20.25] | 0.504 |
| ICU treatments | | | | |
|  | MV use, n (%) | 183 (82.1) | 73 (76.0) | 0.278 |
|  | Days of MV use, median [IQR] | 2.00 [1.00, 3.50] | 2.00 [1.00, 3.00] | 0.353 |
|  | Use of benzodiazepines, n, (%) | 11 (4.9) | 9 (9.4) | 0.139 |
|  | ICU length of stay (days), median [IQR] | 5.00 [4.00, 7.00] | 5.00 [4.00, 7.25] | 0.889 |
| Acute brain dysfunction | | | | |
|  | Days of delirium, median [IQR] | 0.00 [0.00, 1.00] | 0.00 [0.00, 1.25] | 0.759 |
|  | Days of coma, median [IQR] | 0.00 [0.00, 1.00] | 0.00 [0.00, 1.00] | 0.202 |
| Mental health disorder severity and quality of life assessed at one year after ICU discharge | | | | |
|  | HADS anxiety score, median [IQR] | 3.50 [1.00, 6.00] | 5.00 [2.00, 7.00] | 0.031 |
|  | HADS depression score, median [IQR] | 4.67 [2.00, 7.00] | 6.00 [4.00, 9.00] | 0.010 |
|  | IES-R score, median [IQR] | 3.00 [1.00, 8.00] | 4.50 [1.00, 13.00] | 0.172 |
|  | EQ-5D-5L score, median [IQR] | 0.87 [0.74, 1.00] | 0.81 [0.65, 1.00] | 0.040 |

IQR, interquartile range; CV, cardiovascular; CHF/ AMI/ Arrhy, congestive heart failure/ acute myocardial infarction/ arrhythmia; ENT, ear nose throat; APACHE II, Acute Physiology and Chronic Health Evaluation II; MV, mechanical ventilation; ICU, intensive care unit; HADS, Hospital Anxiety and Depression Scale; EQ-5D-5L, EuroQOL 5 Dimensions 5 Level.
