# Supplementary material for: Long-term mental health change patterns in ICU survivors: a four-year comparative follow-up from the SMAP–HoPe study
Source: J Intensive Care. 2025 Jul 28;13:41. doi: 10.1186/s40560-025-00812-z (PMC12302793; doi:10.1186/s40560-025-00812-z)
Supplement: Supplementary file 5 — Additional file 5. Venn diagram for anxiety, depression, and post-traumatic stress disorder in patients 4 years after ICU discharge. [file 40560_2025_812_MOESM5_ESM.docx]

**Additional file 5**


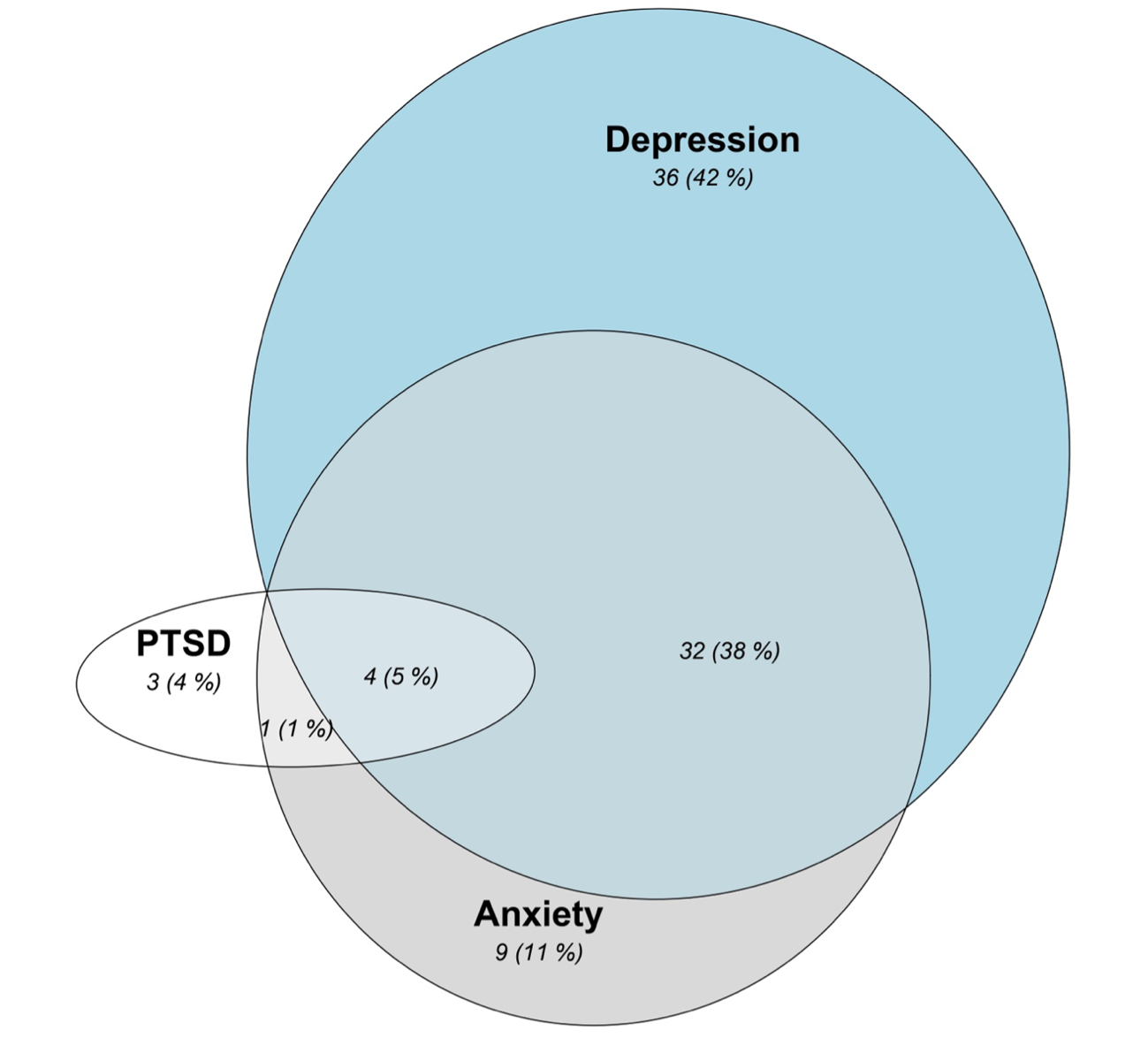


Venn Diagram for anxiety, depression, and post-traumatic stress disorder in patients 4 years after ICU discharge. The Venn diagram depicts the prevalence and comorbidity patterns of three mental health conditions. Depression emerged as the most prevalent condition, with considerable overlap observed between depression, anxiety, and PTSD.

PTSD, post-traumatic stress disorder.
