# Supplementary material for: Long-term mental health change patterns in ICU survivors: a four-year comparative follow-up from the SMAP–HoPe study
Source: J Intensive Care. 2025 Jul 28;13:41. doi: 10.1186/s40560-025-00812-z (PMC12302793; doi:10.1186/s40560-025-00812-z)
Supplement: Supplementary file 6 — Additional file 6. Correlation matrix of symptom changes in anxiety, depression, and PTSD from 1-year to 4-year follow-up. [file 40560_2025_812_MOESM6_ESM.docx]

**Additional file 6**


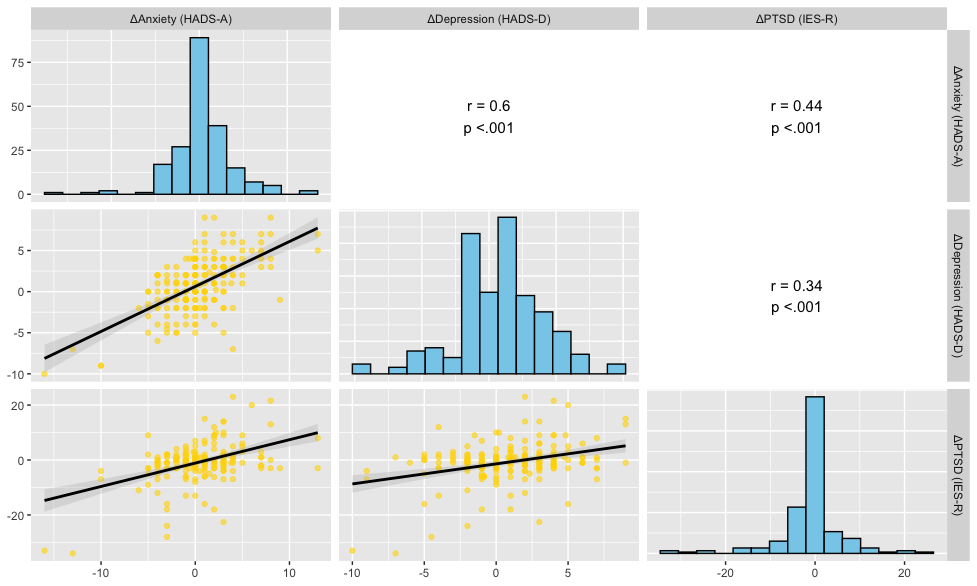


Correlation analysis of changes in mental health symptoms between 1 and 4 years after ICU discharge. The figure shows pairwise correlations between change scores (4-year minus 1-year scores) for anxiety (HADS-A), depression (HADS-D), and PTSD (IES-R) symptoms. The upper triangle displays correlation coefficients and p-values, while the lower triangle shows scatter plots with regression lines and 95% confidence intervals. Histograms along the diagonal represent the distribution of change scores for each symptom domain.

HADS-A, Hospital Anxiety and Depression Scale - Anxiety subscale; HADS-D, Hospital Anxiety and Depression Scale - Depression subscale; IES-R, Impact of Event Scale-Revised; ICU, intensive care unit.
