# Supplementary material for: Long-term mental health change patterns in ICU survivors: a four-year comparative follow-up from the SMAP–HoPe study
Source: J Intensive Care. 2025 Jul 28;13:41. doi: 10.1186/s40560-025-00812-z (PMC12302793; doi:10.1186/s40560-025-00812-z)
Supplement: Supplementary file 7 — Additional file 7. Statistical indices for evaluating growth mixture models in longitudinal analysis of psychological symptom change patterns. [file 40560_2025_812_MOESM7_ESM.docx]

**Additional file 7**

**Statistical Indices for Evaluating Growth Mixture Models in Longitudinal Analysis of Psychological Symptom Change Patterns: A Comparative Study of HADS-A, HADS-D, and IES-R Scales Using Different Covariance Structures**

A three-class model was ultimately determined to be optimal for all three scales after comparing the models with different numbers of classes (1-5) based on statistical criteria and theoretical considerations. For the Hospital Anxiety and Depression (HADS)-A scale measuring anxiety symptoms, we adopted the EVI (equal volume, varying shape, equal shape) model, which allows clusters to have the same volume but different shapes and orientations in the multivariate space (BIC = -2274.81, entropy = 0.646). This model configuration accommodates anxiety symptom patterns with similar overall magnitude but different specific manifestation patterns and directional trends over time. For the HADS-D scale measuring depressive symptoms, the VEE (varying volume, equal shape, equal orientation) model was selected, which allows clusters to have different volumes but the same shape and orientation (BIC = -2454.01, entropy = 0.619). This structure reflects depressive symptom change patterns that maintain similar patterns and directions but differ in their overall severity or magnitude across subgroups.

For the Impact of Event Scale-Revised (IES-R) scale measuring post-traumatic stress symptoms, the VEV (varying volume, equal shape, varying orientation) model provided the best fit, allowing clusters to have different volumes and orientations while maintaining the same shape (BIC = -2653.09, entropy = 0.814). This configuration captures post-traumatic stress change patterns that share a common underlying shape pattern but differ both in overall severity and in their directional evolution over the assessment period.

Among these three models, the IES-R showed both the lowest BIC value (indicating better model fit with respect to parsimony) and the highest entropy value (indicating clearer class separation with values closer to 1.0 representing better classification accuracy), suggesting that it achieved the best statistical fit among the three scales analyzed. The entropy value of 0.814 for the IES-R model indicates particularly good classification precision, with approximately 81.4% of cases being correctly classified into their respective change pattern classes. This superior fit indicates that the three distinct change patterns of post-traumatic stress symptoms were more clearly differentiated compared with the change patterns found in anxiety and depressive symptoms, which showed moderate classification quality with entropy values of 0.646 and 0.619, respectively.
