# Supplementary material for: Long-term mental health change patterns in ICU survivors: a four-year comparative follow-up from the SMAP–HoPe study
Source: J Intensive Care. 2025 Jul 28;13:41. doi: 10.1186/s40560-025-00812-z (PMC12302793; doi:10.1186/s40560-025-00812-z)
Supplement: Supplementary file 8 — Additional file 8. Sensitivity analysis using available case analysis: factors associated with anxiety, depression, and post-traumatic stress disorder change pattern classes. [file 40560_2025_812_MOESM8_ESM.docx]

**Additional file 8**

**Sensitivity analysis using available case analysis: Factors associated with anxiety, depression, and post-traumatic stress disorder change pattern classes**

**Sensitivity analysis using available case analysis: Factors associated with anxiety change pattern classes**

|  | Mild-decreasing (Class 1) vs  Minimum-increasing (Class 2) | | Mild-decreasing (Class 1) vs  Moderate-stable (Class 3) | |
| --- | --- | --- | --- | --- |
|  | aOR (95% CI) | p-value | aOR (95% CI) | p-value |
| Age | 1.01 (0.96, 1.07) | 0.600 | 0.99 (0.96, 1.02) | 0.483 |
| Higher education | 0.42 (0.20, 0.87) | 0.020 | 0.25 (0.10, 0.61) | 0.002 |
| Days of delirium | 1.03 (0.86, 1.24) | 0.100 | 0.93 (0.73, 1.18) | 0.537 |
| APACHE II | 1.14 (0.96, 1.07) | 0.604 | 0.94 (0.88, 1.01) | 0.100 |

Mild-decreasing (Class 1) indicates reference class.

APACHE II, Acute Physiology and Chronic Health Evaluation II; aOR, adjusted odds ratio; CI, confidence interval.

**Sensitivity analysis using available case analysis: Factors associated with depression change pattern classes**

|  | Minimum-stable (Class 1) vs  Mild-increasing (Class 2) | | Minimum-stable (Class 1) vs  Moderate-stable (Class 3) | |
| --- | --- | --- | --- | --- |
|  | aOR (95% CI) | p-value | aOR (95% CI) | p-value |
| Age | 0.99 (0.95, 1.03) | 0.624 | 0.98(0.94, 1.02) | 0.287 |
| Higher education | 0.61 (0.25, 1.47) | 0.274 | 0.27(0.10, 0.75) | 0.012 |
| Days of MV use | 1.17 (0.93, 1.45) | 0.174 | 1.03 (0.82, 1.31) | 0.775 |

Minimum-stable (Class 1) indicates reference class.

aOR, adjusted odds ratio; CI, confidence interval; MV, mechanical ventilation.

**Sensitivity analysis using available case analysis: Factors associated with depression change pattern classes**

|  | Minimum-stable (Class 1) vs  Mild-stable (Class 2) | | Minimum-stable (Class 1) vs  Moderate-stable (Class 3) | |
| --- | --- | --- | --- | --- |
|  | aOR (95% CI) | p-value | aOR (95% CI) | p-value |
| Age | 0.99 (0.96, 1.02) | 0.492 | 0.98 (0.96, 1.04) | 0.297 |
| Male | 0.32 (0.13, 0.80) | 0.014 | 0.41 (0.18, 1.03) | 0.059 |
| Higher education | 2.40 (1.08, 5.39) | 0.032 | 0.65 (0.27, 1.62) | 0.363 |
| Scheduled surgery | 1.40 (0.67, 2.93) | 0.377 | 0.70 (0.33, 1.49) | 0.360 |
| Days of delirium | 1.14 (0.87, 1.50) | 0.345 | 1.21 (0.94, 1.56) | 0.141 |

Minimum-stable (Class 1) indicates reference class.

aOR, adjusted odds ratio; CI, confidence interval.
